# Application of Ni-Oxide@TiO_2_ Core-Shell Structures to Photocatalytic Mixed Dye Degradation, CO Oxidation, and Supercapacitors

**DOI:** 10.3390/ma9121024

**Published:** 2016-12-20

**Authors:** Seungwon Lee, Jisuk Lee, Kyusuk Nam, Weon Gyu Shin, Youngku Sohn

**Affiliations:** 1 Department of Chemistry, Yeugnam University, Gyeongsan 38541, Korea; jamb007@naver.com (S.L.); tnrtnrtnr063@naver.com (J.L.); zxvcod@naver.com (K.N.); 2 Department of Mechanical Engineering, Chungnam National University, Daejeon 34134, Korea

**Keywords:** NiO@TiO_2_, core-shell, photocatalyst, CO oxidation, supercapacitor

## Abstract

Performing diverse application tests on synthesized metal oxides is critical for identifying suitable application areas based on the material performances. In the present study, Ni-oxide@TiO_2_ core-shell materials were synthesized and applied to photocatalytic mixed dye (methyl orange + rhodamine + methylene blue) degradation under ultraviolet (UV) and visible lights, CO oxidation, and supercapacitors. Their physicochemical properties were examined by field-emission scanning electron microscopy, X-ray diffraction analysis, Fourier-transform infrared spectroscopy, and UV-visible absorption spectroscopy. It was shown that their performances were highly dependent on the morphology, thermal treatment procedure, and TiO_2_ overlayer coating.

## 1. Introduction

Core-shell nanostructures have been developed to obtain synergistic effects between core materials and interfacial formations by controlling the shell thickness [1,2,3,4,5,6,7]. Core-shell materials obtained by hybridization of two different materials can provide substantial advantages. Hybridization of Ni oxide (or Ni) and TiO_2_ has been studied and applied to the development of energy storage materials, ultraviolet detectors, CO_2_ reduction, various catalytic reactions such as hydrogen production and CO methanation, and photocatalysis [4,5,6,7,8,9,10,11,12,13,14,15,16,17,18,19]. Various hybridization methods were employed to synthesize materials with diverse morphologies [20], including template-assisted electrodeposition [11], anodizing process [21,22], hydrothermal method [4,6,9], and flame spray pyrolysis [12]. To synthesize three-dimensional (3D) Ni/TiO_2_ nanowires, Wang et al. employed Ni electrodeposition by using a porous anodic alumina template, followed by TiO_2_ coating by atomic layer deposition; notably, they reported substantial increases in areal discharging capacity and rate capability [11]. Kim et al. fabricated NiO-TiO_2_ nanotube arrays (NTAs) by anodizing Ni-Ti foils at potentials in the range of 20–80 V, and used them in supercapacitors [8]. Li-storage performance (areal discharge capacity) and stability (cyclic performance) of 3D NiO nanostructures were found to substantially increase upon loading of TiO_2_ nanoparticles (NPs) [9]. Ke et al. synthesized TiO_2_@Ni(OH)_2_ core-shell nanowire arrays by hydrothermal method and chemical bath deposition, obtaining a supercapacitor with a high capacity of 264 mA·h·g^−1^ [4]. Ni-Ti-O NTAs by electrochemical anodization were also used for catalytic electro-oxidation of methanol [22]. Enhanced photocatalytic degradation of methyl orange (MO) was reported when using NiO-TiO_2_ NTAs [23]. The enhanced efficiency (compared with that of bare TiO_2_ NTAs) was attributed to the interfacial charge transfer of photogenerated electrons from TiO_2_ to NiO [23]. Yu et al. used a hierarchical porous flower-like NiO/TiO_2_ p-n junction for photocatalytic removal of *p*-chlorophenol, and observed remarkable photocatalytic activity as well as cycling stability [24]. Disinfection of bacteria (e.g., *Escherichia coli*) was demonstrated by using NiO-TiO_2_ composite NPs [25]. Chockalingam et al. showed that composite NPs were more efficient than bare TiO_2_ NPs for photocatalytic degradation of phenol [25]. However, their photocatalytic efficiency was poorer than that of bare TiO_2_ for the degradation of rhodamine B (RhB) and methylene blue (MB) [25]. Shinde and Madrad prepared Ni NPs supported on TiO_2_ by low-temperature sonication method, reporting higher stability and activity for the CO methanation reaction which was attributed to the strong metal-support interaction, and created oxygen defects [14]. Ong et al. prepared CNT@Ni/TiO_2_ nanocomposites by coprecipitation and subsequent chemical vapor deposition, reporting high CH_4_ production (from CO_2_) yield of 0.145 μmol·g^−1^·h^−1^ [15]. Gao et al. demonstrated the use of nanoporous NiO/TiO_2_ layers as a glucose sensor with a detection limit of 1.0 μM and a sensitivity of 252.0 μA·mM^−1^·cm^−2^ [26]. Other various TiO_2_-based core-shell structures such as SiO_2_@TiO_2_ microspheres and PS/Au/TiO_2_ nanospheres have been successfully applied for photocatalysis, light trapping, and advanced diagnostics [27,28,29,30,31]. For example, Alessandri prepared SiO_2_@TiO_2_ core-shell microspheres by atomic layer deposition and found a remarkable enhancement of Raman scattering without plasmonic enhancers [29,30].

Although NiO@TiO_2_ and TiO_2_@NiO core-shell materials were reported, until now, thorough studies on the diverse applications of the same materials treated with different methods have not been reported. The novelty of the present study stems from the use of NiO@TiO_2_ core-shells in three different application areas—i.e., photocatalytic dye degradation for water treatment, CO oxidation for air treatment, and supercapacitors for energy storage. In addition, the application efficiency was found to be affected by the thermal treatment procedure. The results obtained here can be very useful for the development of hybrid materials in various fields.

## 2. Results and Discussion

**Figure 1:**
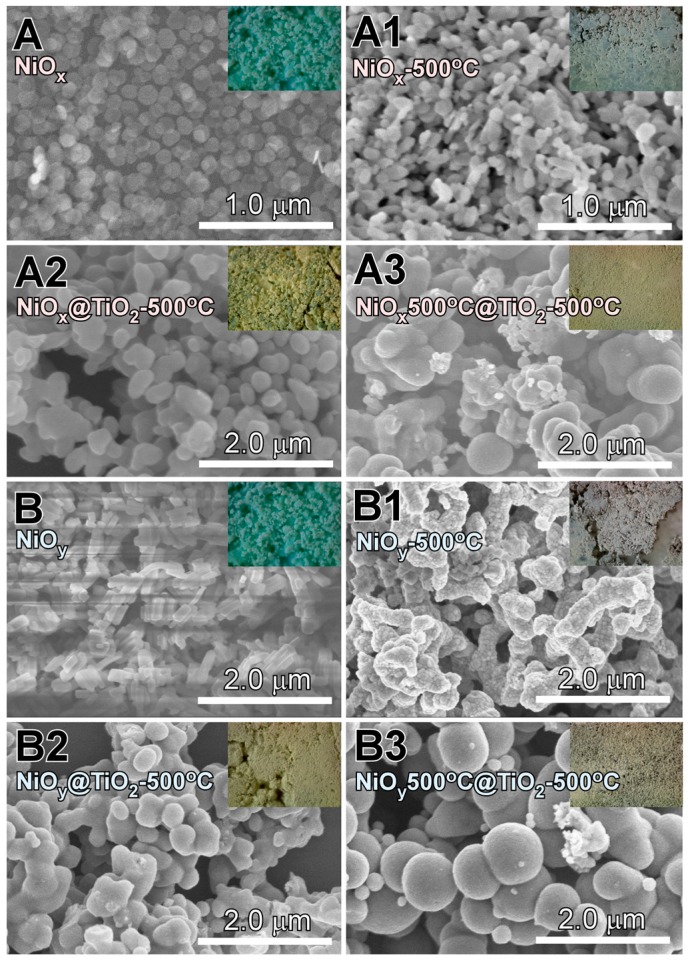
Field-emission scanning electron microscopy (FE-SEM) images of as-prepared NiO (**A** and **B**), A and B after annealing at 500 °C (**A1** and **B1**), TiO_2_-coated A and B followed by annealing at 500 °C (**A2** and **B2**), and TiO_2_-coated A1 and B1 followed by annealing at 500 °C (**A3** and **B3**). The starting materials A and B show different morphologies.

Scanning electron microscopy (SEM) analysis was conducted to examine the sample morphology (Figure 1). The morphologies of the as-synthesized A (NiO*_x_*) and B (NiO*_y_*) samples resembled hexagonal plates and short rods, respectively, while their color appeared bluish-green, which is characteristic of Ni(II) complexes [32,33]. Hexagonal plate morphology was also reported for a Ni-rich Ni-Co complex with green color [33]. When the A and B samples were annealed at 500 °C—denoted as A1 (NiO*_x_*-500 °C) and B1 (NiO*_y_*-500 °C)—the color changed to grey, indicating a modification in crystal structure. In addition, the morphologies underwent some changes, exhibiting particles of various sizes and worm-like structures of 1–2 μm, respectively. In the case of the A2 (NiO*_x_*@TiO_2_-500 °C) and B2 (NiO*_y_*@TiO_2_-500 °C) samples, obtained by coating with TiO_2_ and subsequently annealing (500 °C) the A and B samples, the colors changed to dark yellow. The particle sizes (100–300 nm) of sample A2 were bigger than those of sample A1. Sample B2 showed an aggregated sphere-like morphology, with sizes of 100–400 nm. The samples obtained upon TiO_2_ coating and annealing of the A1 and B1 samples are denoted as A3 (NiO*_x_*500 °C@TiO_2_-500 °C) and B3 (NiO*_y_*500 °C@TiO_2_-500 °C). The A3 and B3 samples had a similar yellowish-grey color as well as a sphere-like morphology with some small particles.

**Figure 2:**
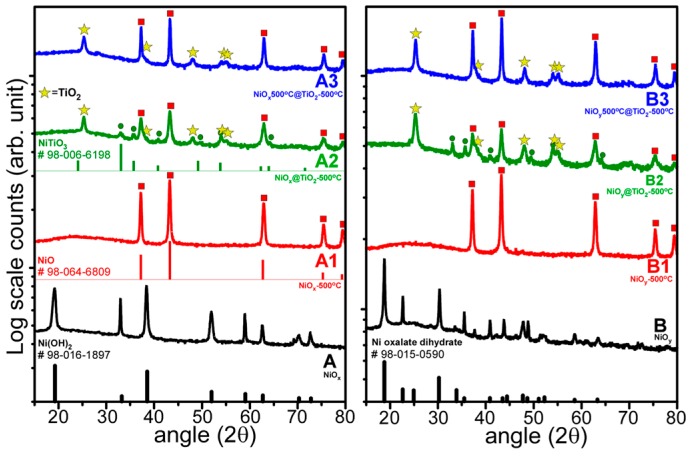
X-ray diffraction (XRD) patterns of as-prepared NiO (**A** and **B**), A and B after annealing at 500 °C (**A1** and **B1**), TiO_2_-coated A and B followed by annealing at 500 °C (**A2** and **B2**), and TiO_2_-coated A1 and B1 followed by annealing at 500 °C (**A3** and **B3**) samples. Reference patterns are also shown at the bottom.

Figure 2 shows the X-ray diffraction (XRD) patterns of the materials in Figure 1. The XRD pattern of sample A matched that of hexagonal Ni(OH)_2_ (reference code: 98-016-1897) [34,35,36,37]. The peaks at 2θ = 19.2°, 33.0°, and 38.5° can be assigned to the (001), (010), and (011) crystal planes of the hexagonal crystal phase, respectively. The XRD pattern of sample B was close to that of monoclinic Ni oxalate dehydrate (reference code: 98-015-0590) with a nearly overlapped strong peak at 2θ = 18.8°, assignable to the (200)/($20\bar{2}$) crystal planes. The A1 (NiO*_x_*-500 °C) and B1 (NiO*_y_*-500 °C) samples had identical XRD patterns matching that of cubic NiO (reference code: 98-064-6098). The three major peaks at 2θ = 37.3°, 43.0°, and 62.9° can be assigned to the (111), (002), and (022) planes of the cubic crystal phase, respectively. The different Ni complexes of Ni hydroxide and oxalate assumed the same NiO structure upon thermal annealing at 500 °C. Cui et al. synthesized Ni(OH)_2_ nanosheets and flower-like microspheres by hydrothermal method, using ammonia and polyvinylpyrrolidone at 150 °C for 15 h, and obtained NiO nanosheets and NiO microspheres by thermally annealing the corresponding samples at 500 °C [34]. For the other four TiO_2_-coated samples (A2, A3, B2, and B3), XRD patterns corresponding to that of tetragonal anatase TiO_2_ (reference code: 98-020-2243) were observed. The strongest peak at 2θ = 25.3° for TiO_2_ corresponds to the (011) plane of the tetragonal phase. For samples A2 (NiO*_x_*@TiO_2_-500 °C) and B2 (NiO*_y_*@TiO_2_-500 °C), additional XRD peaks were observed and attributed to hexagonal Ni(II) titanate NiTiO_3_ (reference code: 98-006-6198). Kim et al. also reported the formation of the NiTiO_3_ phase for thermally annealed NiO-TiO_2_ NTAs at 600 °C [8]. This indicates that the NiTiO_3_ phase is commonly formed by heating NiO and TiO_2_ via NiO + TiO_2_ + heat → NiTiO_3_. Based on the above results, the A2 and B2 samples were NiO@TiO_2_ core-shell structures with interfacial NiTiO_3_, whereas the A3 and B3 samples were NiO@TiO_2_ core-shell structures with no XRD detectable interface species.

**Figure 3:**
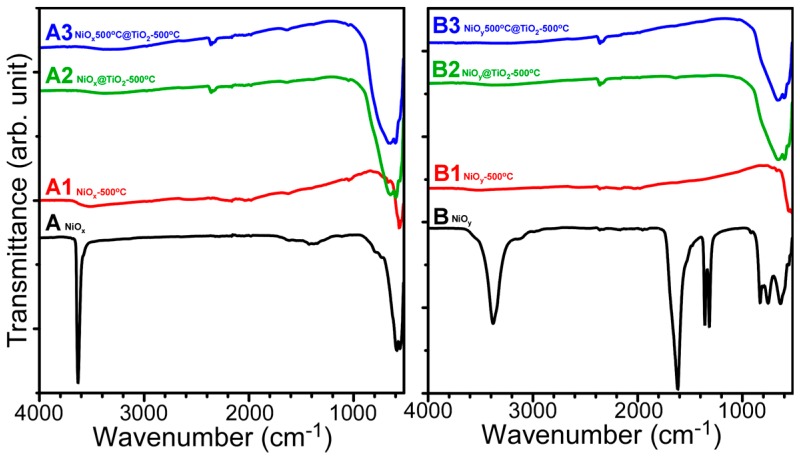
Fourier-transform infrared (FT-IR) spectra of as-prepared NiO (**A** and **B**), A and B after annealing at 500 °C (**A1** and **B1**), TiO_2_-coated A and B followed by annealing at 500 °C (**A2** and **B2**), and TiO_2_-coated A1 and B1 followed by annealing at 500 °C (**A3** and **B3**).

Figure 3 shows the Fourier-transform infrared (FT-IR) spectra for the eight samples in Figure 1. The samples with identical XRD patterns showed similar FT-IR spectra—namely, the A1, A2, and A3 spectra—were similar to the B1, B2, and B3 spectra, respectively. The formation of the Ni oxalate complex of the B sample was more advanced than that of the A sample. Ni–OH stretching vibrations were observed at 3630 and 3380 cm^−1^ for the A and B samples, respectively [33,38]. For samples A1 and B1, the Ni–O vibration peak was observed at ~560 cm^−1^ [33,38]. For the TiO_2_-coated samples, broad peaks were commonly observed at ~650 cm^−1^, and attributed to the Ti–O–Ti vibration of the TiO_2_ lattice [39].

**Figure 4:**
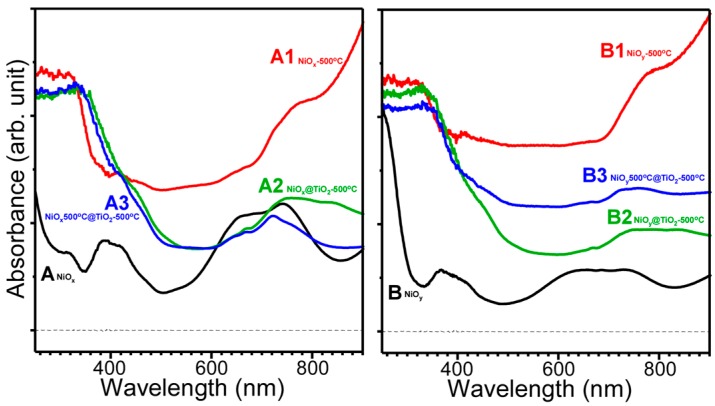
Ultraviolet-visible absorption spectra of as-prepared NiO (**A** and **B**), A and B after annealing at 500 °C (**A1** and **B1**), TiO_2_-coated A and B followed by annealing at 500 °C (**A2** and **B2**), and TiO_2_-coated A1 and B1 followed by annealing at 500 °C (**A3** and **B3**).

Ultraviolet-visible (UV-Vis) absorption spectra (Figure 4) were obtained to further characterize the samples showing different sample colors. For the as-synthesized samples A (NiO*_x_*) and B (NiO*_y_*), two strong absorption regions were observed at ~400 and ~700 nm, attributed to the d-orbital bonding transitions commonly observed for Ni(II) complexes [40]. For the other samples with NiO crystal phase (A1, A2, A3, B1, B2, and B3), broad absorption peaks were observed at ~700–800 nm, which could be attributed to the various transitions (e.g., from the ground state of ^3^A_2g_ to the excited states of ^3^T_2g_ and ^3^T_1g_) of Ni(II) with octahedral coordination [34]. For the NiO@TiO_2_ core-shell structures (A2, A3, B2, and B3), band gap absorption edges were observed near 500 nm.

**Figure 5:**
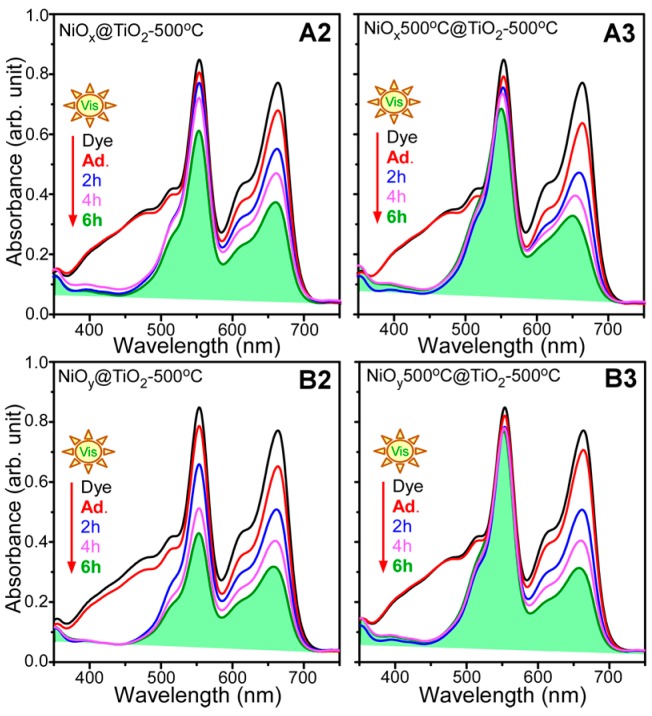
Ultraviolet-visible absorption spectra for photocatalytic mixed dye degradation under visible-light irradiation obtained using as-synthesized NiO followed by TiO_2_ coating and annealing at 500 °C (**A2** and **B2**), and pre-annealed (500 °C) NiO followed by TiO_2_ coating and annealing at 500 °C (**A3** and **B3**).

**Figure 6:**
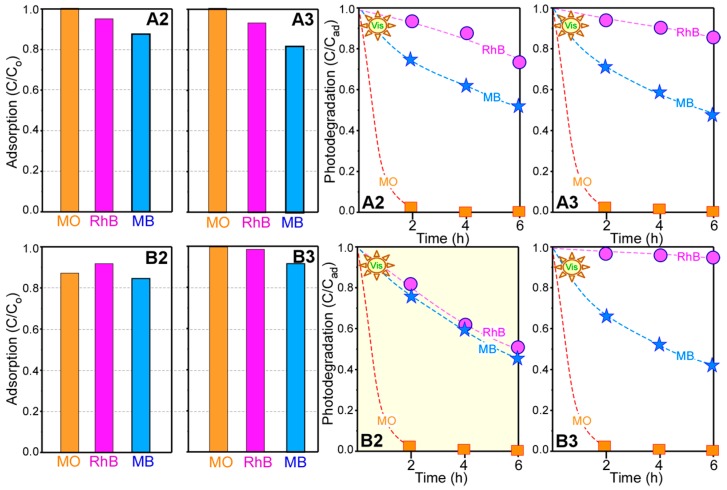
Adsorption (C/C_o_) and photodegradation (C/C_ad_) performance profiles of photocatalytic mixed dye solutions containing as-synthesized NiO followed by TiO_2_ coating and annealing at 500 °C (**A2** and **B2**), and pre-annealed (500 °C) NiO followed by TiO_2_ coating and annealing at 500 °C (**A3** and **B3**). The corresponding ultraviolet-visible (UV-Vis) absorption spectra are shown in Figure 5. C_0_ and C_ad_ indicate the UV-Vis absorption intensities of the mixed solution before and after adsorption, respectively.

As TiO_2_ is widely used in photocatalysis, the NiO@TiO_2_ core-shells (A2, A3, B2, and B3) were tested for photocatalytic dye degradation [41,42,43,44,45,46,47,48,49,50,51]. To increase the novelty of this work, as pure dyes have been extensively investigated, we selected rarely studied mixed dyes, which are also more practical [41,42,43,44,45]. Figure 5 shows the UV-Vis absorption spectra of mixed dye solutions containing dispersions of A2 and B2 samples, and A3 and B3 samples, at increasing visible-light (>400 nm) exposure times. The mixed dye solution was a mixture of MO (5 ppm = 5 mg/L), RhB (5 ppm), and MB (5 ppm); its UV-Vis spectrum showed three absorption peaks at 450, 550, and 650 nm, corresponding to the absorption centers of MO, RhB, and MB, respectively [41,42,43,44,45]. The three peak positions were selected to analyze the degradation rate of each dye. Figure 6 displays the analyzed data for the corresponding UV-Vis absorption spectra in Figure 5. The UV-absorption spectra (Figure 5) were taken upon achieving adsorption-desorption equilibrium for 1 h under dark conditions. MO was negligibly adsorbed on the A2, A3, and B3 samples, while 15% of MO was adsorbed on the B2 sample (Figure 6). MB showed relatively good adsorption. Adsorption performance has commonly been explained by electrostatic interactions between the dyes and the catalysts surface [49,50]. Because MB and RhB molecules are more positively charged than MO, the negatively surface-charged catalyst will adsorb MB and RhB more strongly than MO. For the increased adsorption of MO for B2 sample, the combination of NiO/NiTiO_3_/TiO_2_ could govern the surface interactions between MO and the B2 sample surface. The order of dye adsorption was found to be MO < RhB < MB for the A2, A3, and B3 samples, and RhB < MO < MB for the B2 sample. The B2 sample showed the best adsorption performance. More interesting results were observed by increasing the visible-light exposure time. Among the three dyes, MO was the fastest to degrade, showing a similar behavior on all the samples. The degradation rate typically showed the following order: RhB < MB << MO. In addition, MB exhibited similar degradation in all the samples; conversely, RhB showed drastic differences between the different samples. The photocatalytic activity for RhB showed an order of B3 << A3 < A2 < B2. The B3 sample showed negligible photocatalytic activity for RhB, while the B2 sample showed a good catalytic activity, comparable to that of MB. The A2 sample showed better activity than the A3 sample for photocatalytic RhB degradation. It appeared that the interfacial NiTiO_3_ phase formation (for A2 and B2) facilitated the RhB degradation. For all three dyes, the B2 sample showed the best catalytic performance for mixed dye degradation. Some studies on catalysts for the photocatalytic degradation of mixed dyes are summarized in Table 1 [41,42,43,44,52].

As the dyes and samples absorb visible light, photogenerated electron hole pairs can be formed by direct visible-light absorption by mixed dye and NiO@TiO_2_. The electron (e^−^) and hole (h^+^) pairs are separated by interfacial charge transfer at the interface of the p-n junction [5,6,18,46]. The electrons in the mixed dye can transfer to the conduction band (CB) of NiO@TiO_2_; they are then captured by oxygen to generate active •O_2_^−^ (or •O^−^), which can further react with H^+^ to form •O_2_H (or •OH). Consequently, the active species of •O_2_^−^, h^+^, and •OH are used for dye degradation. The summarized mechanism is written below [47,48,49,50,51]:

Mixed dye + visible light → mixed dye (e^−^_CB_ + h^+^_VB_)

NiO@TiO_2_ + visible light → NiO@TiO_2_ (e^−^_CB_ + h^+^_VB_) and charge separation at the interface

Adsorbed mixed dye (e^−^_CB_ + h^+^_VB_) + NiO@TiO_2_ → NiO@TiO_2_ (e^−^_CB_) + Mixed dye (h^+^_VB_)

NiO@TiO_2_ (e^−^_CB_) + adsorbed or surface O_2_ (or O) → •O_2_^−^ (or •O^−^) + NiO@TiO_2_

•O_2_^−^ (or •O^−^) + H^+^ → •O_2_H (or •OH)

H_2_O + NiO@TiO_2_ (h^+^_VB_) → H^+^ + •OH

•O_2_^−^, h^+^, and •OH + adsorbed mixed dye/dye^+^ → degradation products

**Figure 7:**
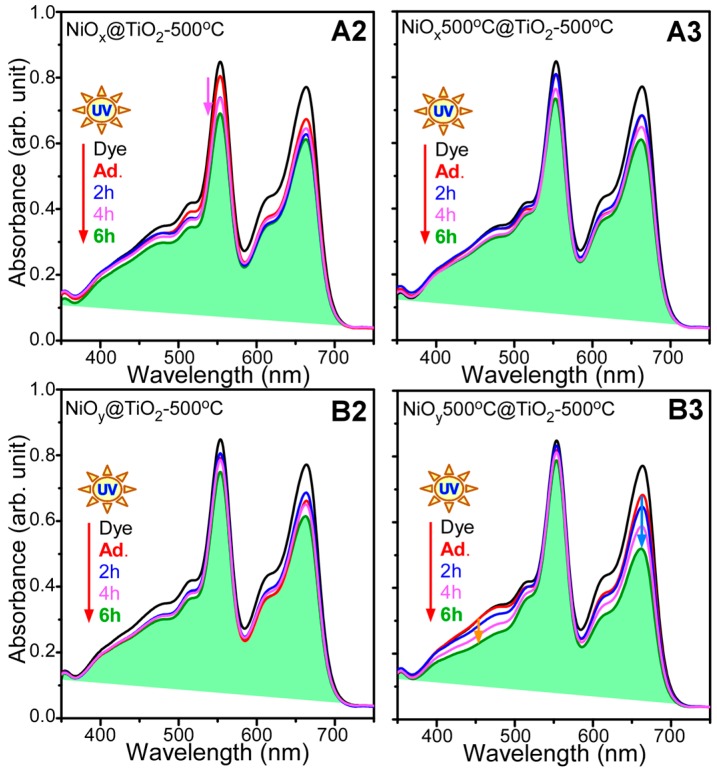
Ultraviolet (UV) visible absorption spectra for photocatalytic mixed dye degradation under UV-light irradiation obtained using as-synthesized NiO followed by TiO_2_ coating and annealing at 500 °C (**A2** and **B2**), and pre-annealed (500 °C) NiO followed by TiO_2_ coating and annealing at 500 °C (**A3** and **B3**).

Photocatalytic mixed dye degradation under UV light (365 nm) was also tested (Figure 7). Unlike under visible-light conditions, the photocatalytic activity showed different behavior under UV-light conditions. MO did not show the fastest degradation rate. The A2 sample showed better photocatalytic activity for RhB than the other samples. The B3 sample showed better photocatalytic activity for MO and MB than the other samples. The B2 sample showed the poorest photocatalytic activity for the mixed dye solution under UV light (Figure 7); however, it showed the best photocatalytic activity under visible light (Figure 5 and Figure 6). Based on these results, the photocatalyst selection depends on the light wavelength.

**Figure 8:**
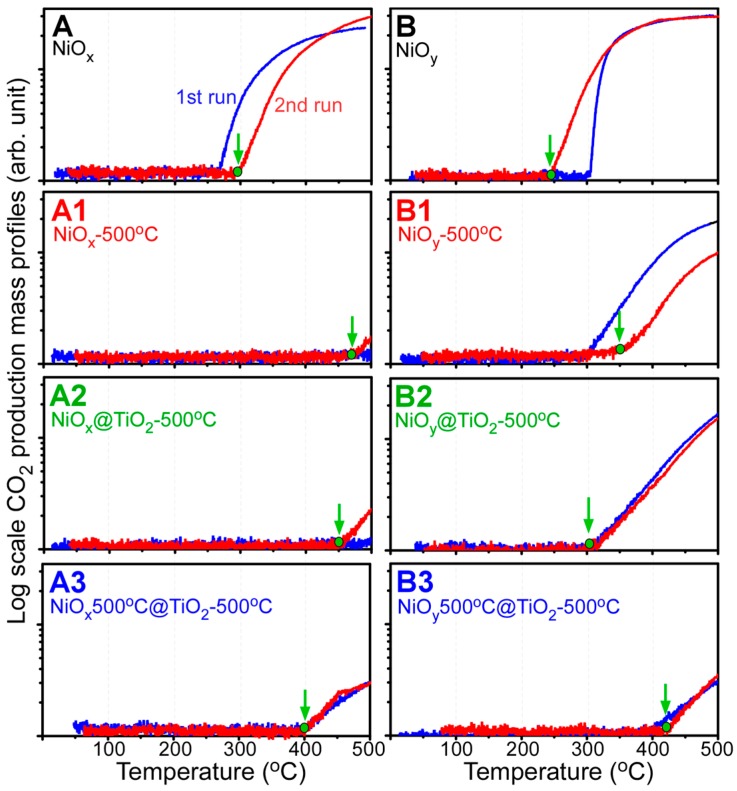
CO_2_ production (by CO oxidation) mass profiles for as-prepared NiO (**A** and **B**), annealed (500 °C) A and B (**A1** and **B1**), TiO_2_-coated A and B followed by annealing at 500 °C (**A2** and **B2**), and TiO_2_-coated A1 and B1 followed by annealing at 500 °C (**A3** and **B3**).

The materials were tested as CO oxidation catalysts. Figure 8 displays the first and second CO oxidation reaction run profiles. CO_2_ (mass = 44 amu) production by CO oxidation was monitored with the increasing reaction temperature by using mass spectrometry [33,53,54,55,56]. Qualitative analysis was performed using the mass profile data. For the first runs of the as-synthesized samples—i.e., A (NiO*_x_*) and B (NiO*_y_*)—the CO_2_ production onsets were observed at 265 and 300 °C, respectively; while, in the second runs, they were observed at 300 and 250 °C, respectively. In the second run, sample A showed degraded activity (by +35 °C), whereas sample B showed enhanced activity (by −50 °C). The Ni hydroxide (sample A) and Ni oxalate (sample B) complexes were found to change to the same NiO crystal phase after the CO oxidation reactions, based on the XRD results. For sample A1, the onsets were observed above 470 °C in the first and second CO oxidation runs. The catalytic activity dramatically degraded for sample A1 with NiO crystal phase. For sample B1, the onsets were observed at 300 and 350 °C in the first and second runs, respectively. Although the B1 sample showed the same NiO crystal phase, its catalytic activity was considerably superior to that of sample A1. Similarly, samples A2 and B2 showed an onset above 450 °C and at 300 °C, respectively. Samples A3 and B3 showed similar onsets near 400 °C for the first and second runs. As a result, the sample preparation method is crucial to determine the catalytic activity. In the present study, the TiO_2_ coating on the B2 sample did not to degrade the CO oxidation activity. However, when Ni(OH)_2_ plates were used as starting material, the CO oxidation activity significantly decreased.

**Figure 9:**
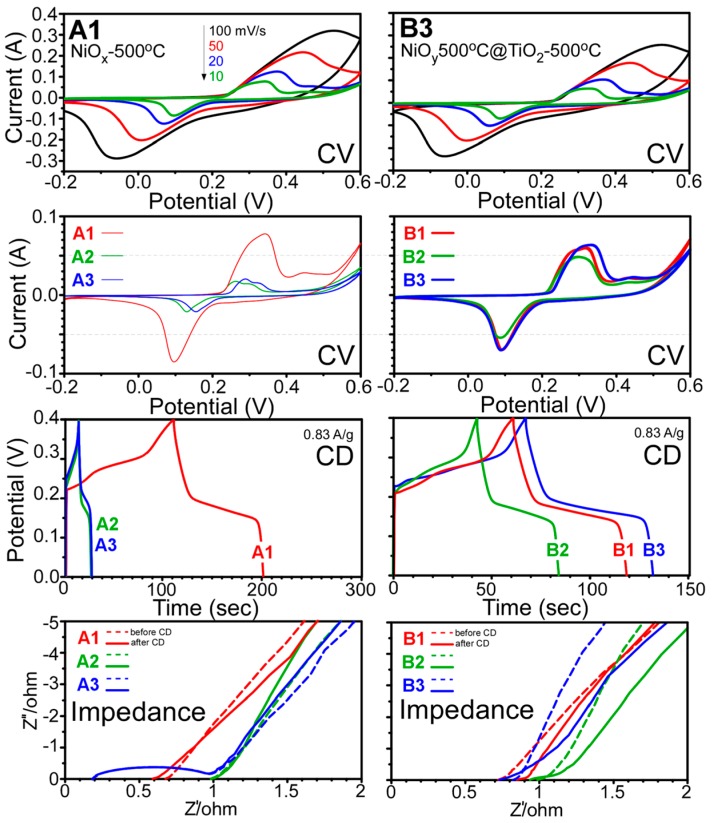
Cyclic voltammetry curves (voltage range: −0.2~0.6 V) at different scan rates (10, 20, 50, and 100 mV/s), charge-discharge curves at the current density of 0.83 A/g, and Nyquist impedance plots for annealed (500 °C) NiO*_x_* and NiO*_y_* samples (**A1** and **B1**), TiO_2_-coated as-synthesized NiO*_x_* and NiO*_y_* samples followed by annealing at 500 °C (**A2** and **B2**), and A1 and B1 samples followed by TiO_2_ coating and annealing at 500 °C (**A3** and **B3**).

Furthermore, supercapacitor performance tests were briefly performed. The related results of cyclic voltammograms (CVs), galvanostatic charge/discharge curves (CD), and impedance plots are shown in Figure 9. For the selected samples (A1 and B3), cyclic voltammetry curves were obtained at various scan rates. Broad anodic and cathodic peaks were typically observed, associated with the redox reactions of NiO + OH^−^ → NiOOH + e^−^ + H_2_O [33,57]. The other samples showed similar qualitative behaviors. The symmetrical peaks indicated reversible redox reactions. The peak current increased with the increasing scan rate, and the gap between the anodic and cathodic peak positions became wider, revealing a diffusion-controlled pseudocapacitive behavior of the material [4,22,57]. The galvanostatic CD curves were obtained at the current density of 0.83 A/g. The measured specific capacitance (F/g) was found to be in the following order: A2 ≈ A3 < B2 < B1 < B3 < A1. For sample A1 with NiO crystal phase, the specific capacitance was measured to be 320 F/g, while, the specific capacitances of the other two TiO_2_-coated samples (A2 and A3) were found to be identical, 50 F/g. For sample B1 with NiO crystal phase, the specific capacitance was measured to be 189 F/g, lower than that of the A1 sample (320 F/g). However, the other two TiO_2_ coated samples, B2 and B3, exhibited values of 211 F/g and 134 F/g, respectively, which were higher than those of the A1 and A2 samples (50 F/g). Kim et al. reported specific capacitances in the range of 40–100 F/g for NiO-TiO_2_ NTAs and 120–300 F/g for NiO-TiO_2_ nanotube films [8], very close to the values observed in the present study. Some literature values for TiO_2_–NiO hybrid materials are summarized in Table 2, where TiO_2_ was mainly core [4,7,8,58,59]. To the best of our knowledge, the specific capacitance for NiO@TiO_2_ core-shell structures has not been reported. The high-frequency region of the impedance Nyquist plots with real (Z′) and imaginary (Z′′) parts are shown in Figure 9. Ascending straight lines were commonly observed (not shown here) for all the samples in the low-frequency region, corresponding to Warburg diffusion resistance [33,53]. Sample A3 clearly showed a semicircle before and after CD measurements, corresponding to charge transfer resistance. The other samples showed no clear semicircles. The interfacial resistance of the A1 sample was smaller than those of the other two samples (A2 and A3). The resistance of the B2 sample was larger than those of the B1 and B3 samples. Thus, the resistance in the impedance plots was found to be consistent with the order of the measured specific capacitance values.

## 3. Experimental Section 

### 3.1. Synthesis of Ni Oxide and TiO_2_ Coating

Two different Ni oxide complexes—i.e., NiO*_x_* (A) and NiO*_y_* (B)—were synthesized by hydrothermal method as described below, and then used as starting materials. For material A, 0.475 g of NiCl_2_·6H_2_O (GR 98%, Duksan Pure Chemical Co., Ansan, Korea) was dissolved in 40 mL of deionized water, and 2 mL of 1.0 M NaOH solution was added to it. The solution was then transferred to a Teflon-lined stainless autoclave and placed in an oven setting at 120 °C for 12 h. For material B (NiO*_y_*), 0.951 g of NiCl_2_·6H_2_O was dissolved in a mixed solvent (20 mL of H_2_O + 20 mL of ethylene glycol); then, 1.0 mL of 0.1 M NaOH solution and 0.238 g of oxalic acid (≥99%, Sigma-Aldrich, St. Louis, MO, USA) were added to it. Upon complete dissolution, the entire solution was transferred to a Teflon-lined stainless autoclave and placed in an oven setting at 120 °C for 12 h. After the hydrothermal reaction, the obtained precipitates were fully washed with deionized water and ethanol, and then centrifuged, repeatedly. The washed sample powders were dried in an oven at 70 °C for two days. For materials A1 (NiO*_x_*-500 °C) and B1 (NiO*_y_*-500 °C), the dried sample powders were thermally treated at 500 °C in an electric furnace for 4 h. For materials A2 (NiO*_x_*@TiO_2_-500 °C) and B2 (NiO*_y_*@TiO_2_-500 °C), the dried A and B sample powders (0.1 g) were dispersed in ethanol solvent, and 0.5 mL of titanium (IV) isopropoxide (TTIP; 97%, Sigma-Aldrich, St. Louis, MO, USA) was added to it. While stirring the solution, water vapor was slowly introduced using a humidifier for 2 h. After the reaction, the samples were fully washed with ethanol solvent to remove uncoated TTIP. Then, the collected sample was dried and thermally annealed at 500 °C for 4 h. For materials A3 (NiO*_x_*500 °C@TiO_2_-500 °C) and B3 (NiO*_y_*500°C@TiO_2_-500°C), the A1 and B1 sample powders were used for TiO_2_ coating. The other procedures were the same as those used for the A2 and B2 samples.

### 3.2. Characterization of the Materials

The morphology of the prepared samples was examined using field-emission SEM (FE-SEM, Hitachi SE-4800, Tokyo, Japan). The samples were placed on an HF-etched Si substrate. The crystal phases were identified using powder XRD; the patterns were obtained by using a PANalytical X’Pert Pro MPD diffractometer (PANalytical Inc., Westborough, MA, USA) equipped with a Cu Kα radiation source. FT-IR spectra were measured using a Nicolet iS 10 FT-IR spectrometer (Thermo Scientific, West Palm Beach, FL, USA) with an attenuated total reflection mode between 500 and 4000 cm^−1^. UV-Vis absorption spectra of the samples were recorded using a Neosys-2000 double beam UV-Vis spectrometer (Scinco, Seoul, Korea).

### 3.3. Photocatalytic Dye Degradation, CO Oxidation, and Supercapacitor Performance Tests

For photocatalytic mixed dye degradation, equal amounts of three different dye (i.e., MO, RhB, and MB) solutions with concentrations of 5 mg/L (=ppm) were mixed to prepare a mixed dye test solution. Then, 20 mg of sample powder was dispersed in 50 mL of the mixed dye solution to achieve adsorption-desorption equilibrium under dark conditions for 1 h. Upon achieving the equilibrium, 2 mL of the solution was taken and centrifuged to remove the residual powder, and a UV-Vis absorption spectrum was recorded by using a V-530 UV-Vis spectrometer (Jasco, Tokyo, Japan). As the equilibrium was reached, the mixed dye solution was examined under UV (or visible) irradiation. For visible light irradiation, a 500 W Halogen lamp (>400 nm) was used and the distance from the lamp and the dye solution in 100 mL beaker (a diameter of 5.5 cm) was fixed at about 30 cm. For UV light irradiation, four 6W UV (365 nm) lamps were used. Every 2 h, 2 mL of the solution was collected to record the UV-Vis absorption spectrum. For the CO oxidation reaction, 10 mg of sample powder was loaded in a flow-type quartz U-tube with an inner diameter of 4 mm. CO oxidation experiments were performed by increasing the temperature up to 500 °C at the rate of 10 °C·min^−1^ under flow of CO (1%) and O_2_ (2.5%) mixed gas in N_2_ balance. The reaction gas products, such as CO_2_ (mass = 44 amu), were examined using an SRS RGA200 quadrupole mass spectrometer (Stanford Research System, Sunnyvale, CA, USA). After the first CO oxidation run, the sample was naturally cooled to room temperature; then, the second CO oxidation run was performed. The electrochemical measurements were conducted using a CHI 660D electrochemical work station (CH Instruments, Austin, TX, USA) with a conventional three-electrode configuration (Ag/AgCl reference electrode, Pt wire counter electrode, and sample mounted on a Ni-foam working electrode) in 6.0 M KOH electrolyte solution. For the working electrode, the sample powder (60 wt %) was fully mixed with acetylene black (20 wt %) and poly(vinylidene fluoride) (20 wt %) in 2 mL of *N*-methyl-2-pyrrolidone solvent. The dispersion was slowly dropped onto Ni foam (1 × 1 cm^2^), dried, and pressed to fabricate a thin Ni foam sheet. The CVs were obtained between −0.2 and 6.0 V, galvanostatic CD experiments were conducted with potentials ranging from 0.1 to 0.4 V at the charge density of 0.83 A·g^−1^, and electrochemical impedance measurements were performed over a frequency range from 0.1 MHz to 0.01 Hz.

## 4. Conclusions

In this work, we investigated the preparation method, characteristics, and various application performances of NiO@TiO_2_ core-shell nanostructures. Generally, TiO_2_ coating on Ni(OH)_2_ plates and on annealed NiO showed poor photocatalytic, thermocatalytic, and supercapacitor performances. Therefore, it is advisable to use rod-shaped Ni oxalate complex as a starting material for TiO_2_ coating. The main findings are as follows:

1. Nanoplates and nanorods were synthesized by hydrothermal method, and showed XRD patterns of Ni(OH)_2_ and Ni oxalate complexes, respectively. NiO crystal phase was commonly obtained by thermal annealing at 500 °C.
2. NiO@TiO_2_ core-shells with interfacial NiTiO_3_ could be prepared by TiO_2_ coating on as-synthesized Ni samples, followed by thermal annealing at 500 °C. TiO_2_ coating on annealed (500 °C) NiO, followed by thermal annealing at 500 °C, showed no XRD detectable NiTiO_3_ at the interface.
3. For photocatalytic dye degradation, TiO_2_ coating on Ni oxalate complex followed by thermal annealing (sample B2) showed the best photocatalytic activity for mixed dye degradation under visible light.
4. For CO oxidation, the B2 sample (NiO*_y_*@TiO_2_-500 °C) was also more efficient for CO oxidation than other samples.
5. For the case of specific capacitance, the B3 sample (NiO*_x_*500°C@TiO_2_-500°C) was more efficient for specific capacitance than other samples. Specific capacitances were obtained to be in the range of 50–320 F/g. When Ni(OH)_2_ plates were used as starting material, the TiO_2_ coating showed a specific capacitance of only 50 F/g. However, when rod-shaped Ni oxalate complex was used, the TiO_2_ coating showed specific capacitance values of 211 and 134 F/g.

Briefly, the present study showed that the sample preparation procedures and the annealing steps vary greatly in the application fields to achieve a higher performance. The obtained valuable information on the preparation methods of the investigated core-shell structures could be extended to other transition metal oxide core-shell structures, promoting their development and diverse applications.

## Figures and Tables

**Table 1:** Literature catalysts for the photocatalytic degradation of mixed dyes.

Catalysts	Order of Dye Degradation under Visible Light	Reference
ZnO, ZnS, Au-ZnS, Ag-ZnS	RhB < MB << MO	41
BiOX, AgX/BiOX (X = Cl, Br, I)	RhB < MB << MO	42
TiO_2_/BiOX (X = Cl, Br, I)	RhB < MB << MO	43
Graphene, Charcoal, ZnO, and ZnS/BiOX (X = Cl, Br, I)	RhB < MB < MO	44
Ag/ZnO by wet-milling method	MB < RhB < MO	52
NiO@TiO_2_ core-shells	RhB < MB << MO	This study

**Table 2:** Literature specific capacitance for TiO_2_–NiO hybrid materials

Samples	Preparation Methods	Specific Capacitance	Reference
TiO_2_@Ni(OH)_2_ nanowire arrays	Hydrothermal synthesis and chemical bath deposition	181 F/g at 5 mV/s	4
TiO_2_/NiO nanorod arrays	Hydrothermal synthesis and electro-deposition methods	611 F/g at 2 A/g	7
flower-like NiO–TiO_2_ nanocomposite	One (or multi)-cycle alternate electrodeposition-oxidation and thermal dehydration	46.3 mF·cm^−2^	58
NiO*_x_* decorated TiO_2_ nanotubes	Cyclic voltammetry electrochemical deposition process	689.28 F/g at 1.5 A/g	59
NiO-TiO_2_ nanotubes	Electrochemical anodization and thermal annealing	40–300 F/g	8
NiO@TiO_2_ core-shells	Wet chemical and thermal annealing	50–211 F/g at 0.83 A/g	This study
